# Failure mechanism of hollow tree trunks due to cross-sectional flattening

**DOI:** 10.1098/rsos.160972

**Published:** 2017-04-12

**Authors:** Yan-San Huang, Fu-Lan Hsu, Chin-Mei Lee, Jia-Yang Juang

**Affiliations:** 1 Department of Forestry, National Chung Hsing University, 145 Xingda Road, South District, Taichung City 402, Taiwan, Republic of China; 2 Division of Forest Chemistry, Taiwan Forestry Research Institute, 53 Nanhai Road, Taipei 10066, Taiwan, Republic of China; 3 Division of Forest Utilization, Taiwan Forestry Research Institute, 53 Nanhai Road, Taipei 10066, Taiwan, Republic of China; 4 Department of Mechanical Engineering, National Taiwan University, Taipei 10617, Taiwan, Republic of China

**Keywords:** bending failure, Brazier moment, tangential crack, hollow trunk, orthotropic material, Taiwan red cypress

## Abstract

Failure of hollow trees in urban areas is a worldwide concern, and it can be caused by different mechanisms, i.e. bending stresses or flattening-related failures. Here we derive a new analytical expression for predicting the bending moment for tangential cracking, and compare the breaking moment of various failure modes, including Brazier buckling, tangential cracking, shear failure and conventional bending failure, as a function of *t*/*R* ratio, where *t* and *R* are the trunk wall thickness and trunk radius, respectively, of a hollow tree. We use Taiwan red cypress as an example and show that its failure modes and the corresponding *t*/*R* ratios are: Brazier buckling (Mode I), tangential cracking followed by longitudinal splitting (Mode II) and conventional bending failure (Mode III) for 0 < *t*/*R* < 0.06, 0.06 < *t*/*R* < 0.27 and 0.27 < *t*/*R* < 1, respectively. The exact values of those ratios may vary within and among species, but the variation is much smaller than individual mechanical properties. Also, shear failure, another type of cracking due to maximum shear stress near the neutral axis of the tree trunk, is unlikely to occur since it requires much larger bending moments. Hence, we conclude that tangential cracking due to cross-sectional flattening, followed by longitudinal splitting, is dominant for hollow trunks. Our equations are applicable to analyse straight hollow tree trunks and plant stems, but are not applicable to those with side openings or those with only heart decay. Our findings provide insights for those managing trees in urban situations and those managing for conservation of hollow-dependent fauna in both urban and rural settings.

## 1. Introduction

Hollow tubes have the advantages of resisting bending and torsional moments with a relatively lower weight per unit length than solid cylinders of the same weight [1]. Therefore, tubular structures have merits of a high strength-to-weight ratio and low weight per unit length, resulting in lower material costs. These hollow structures are ubiquitous in nature, such as bamboo stems, cereal stalks, decayed hollow tree trunks [2] and animal bones [3], as well as in artificial structures, such as buildings, bridge frames, athletic halls and aircraft fuselages. In assessing the hazards of old trees on roadsides and in parks, the hollowness is usually inspected by Resistograph^®^, a sensitive drilling-resistance measuring method and/or sonic tomography [4–6]. According to visual tree assessment (VTA) field observations by Mattheck *et al*. [7], a trunk wall thickness *t* to outer radius *R* ratio *t*/*R* of 0.3 is widely used as a criterion for diagnosing a risky tree. However, there is discrepancy between the VTA field observations and the critical trunk wall thickness for failure predicted by classic bending theory, which assumes that the cross section does not ovalize. More factors such as tangential cracking and orthotropic material properties must be taken into consideration. Understanding the mechanism is important from both safety and conservation perspectives. Natural tree hollows are valuable and often essential for many wildlife species. Hollow-bearing trees are being removed at a faster rate than hollows form in many parts of the world (urban and rural) [8]. There are ongoing declines in abundance of fauna dependent on hollow trees for survival. Many hollow trees are cut down from a fear that they may fall at some point in the future and injure someone. Yet many hollow trees are clearly structurally sound as they remain in place for decades. Our aim is to establish a theoretical framework for assessing safety that might result in retention of more hollow trees.

Tree trunks are subject to two kinds of loading, self-weight and wind load, either of which can result in mechanical damage or failure. Self-weight results from gravitational forces acting on the mass of tree, whereas wind load is the result of wind-induced drag forces acting on the crown and trunk. The axial compressive stress due to self-weight usually has a minor effect except for highly slanted trunks in which self-weight can induce large bending stresses due to the horizontal offset of the crown centroid from trunk base. By contrast, strong wind can significantly affect a tree's stability. Wind load acts on the tree crown and causes large bending moments on the trunk and root plate. These moments are the main sources of trunk failure and root overturning.

**Figure 1.**
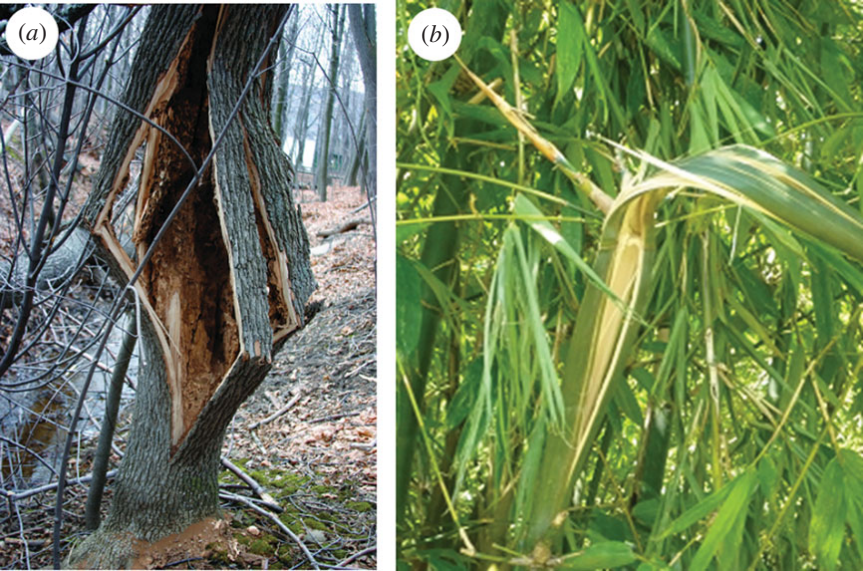
Examples of failure of hollow stems due to cross-sectional flattening. (*a*) Tree trunk split in the grain direction (longitudinal splitting) before buckling; white ash (*Fraxinus americana*) *t*/*R* ≈ 0.1, reproduced from Bond [10] with written permission. (*b*) Bamboo (*Bambusa oldhamii*) stalk split, *t*/*R* ≈ 0.2.

The breaking of thick-walled hollow tree trunks subjected to gale gusts at the crown usually results from bending stresses. Failure begins with fibre buckling on the compression side, followed by fibre tearing on the tension side. In this case, the shape of the cross section may remain almost unchanged until failure occurs, and classic bending theory can be used to calculate the bending strength of long, hollow trunks. But for thin-walled trunks, breaking can be due to cross-section flattening [7,9,10]. Figure 1 illustrates hollow-trunk failure of white ash (*Fraxinus americana*) due to cross-sectional flattening and flattening failure of bamboo (*Bambusa oldhamii*). When an elastic straight beam is subjected to a bending moment, it deforms and becomes a curve in the longitudinal direction. As a result of the deformation, strain on the convex side of the beam is extensive while that on the concave side is compressive. In this case, fibres on the convex side are under tensile stresses and those on the concave side are under compressive stresses; these stresses increase proportionally with the distance from the neutral axis which passes through the centroid of the cross section. Owing to the formation of a curve, transverse compressive forces occur on the convex and concave sides of a beam [11,12]. If the beam is hollow and thin-walled, these forces can ovalize the cross section and cause cracking before buckling. When ovalization occurs, both the moment of inertia and stiffness of the cross section decrease. Ultimately, the cross section collapses and buckles under a smaller moment than would occur due to pure bending stresses. As the cross section flattens, the curvature increases, and the bending moment reaches a maximum value, known as the Brazier moment [13,14]. Spatz & Niklas successfully used numerical simulations to predict the critical bending moments of various failure modes, including bending failure, tangential cracking failure due to ovalization, and Brazier buckling, as a function of the *t*/*R* ratio, slenderness and orthotropic material properties [2]. Although powerful, numerical simulations can sometimes come at the detriment of physical insight and predictive understanding of the interplay between key parameters. Here, we derive an analytical expression, using Castigliano's theorem [15], a strain energy method useful in the treatment of statically indeterminate problems, for predicting the bending moment at which the tangential cracking occurs, and provide a comprehensive comparison on the failure modes for 0 < *t*/*R <* 0.3. Direct calculation of the moments is convenient in assessing the stability of thin-walled hollow trunks, which is appealing from both safety and conservation perspectives.

## 2. Material and methods

### 2.1. Analysis of failure modes

In this section, we determine the failure mode at a given *t*/*R* ratio by comparing the magnitudes of the conventional bending moment (*M*_b_), Brazier breaking moment (*M*_B_) and tangential cracking moment (*M*_c_). The expressions for *M*_b_ and *M*_B_ are obtained from the literature, and the expression for *M*_c_ derived in §2.1.3 is new and has not been reported before.

#### 2.1.1. Failure by conventional bending (*M*_b_)

**Figure 2.**
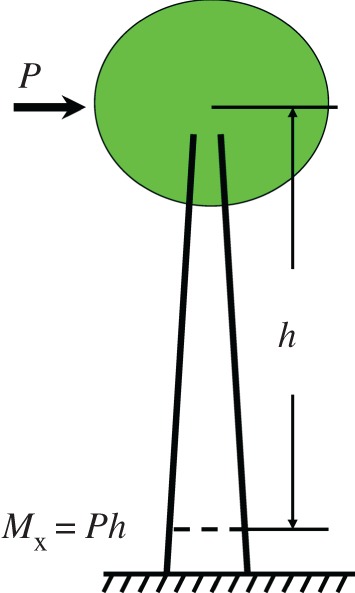
Schematic of a tree loaded with a wind force *P* at the centroid of its crown, and the corresponding internal bending moment *M*_x_ at a distance *h* from the centroid.

Figure 2 illustrates a tree loaded with a wind load *P* at the centroid of its crown. The bending moment *M*_x_ at a distance *h* from the centroid is *Ph*. Assuming that the cross section of a tree trunk is circular, according to classical bending theory the maximum bending moment of a hollow tree trunk *M*_b_ is

2.1 $$M_{b}=\frac{\pi R^{3}\sigma_{b}}{4}\left[1-{\left(1-\frac{t}{R}\right)}^{4}\right],$$

where *σ*_b_ is the bending strength of typical green wood in the longitudinal direction. For small *t*/*R*, $M_{b}=\pi R^{3}(t/R)\sigma_{b}$ and for a solid trunk (*t*/*R* = 1), the bending moment is $M_{b\_\mathrm{solid}}=\pi R^{3}\sigma_{b}/4$.

When bent, the side of tube furthest from the centre of curvature is in tension, and the other side is in compression. If the tensile stress exceeds the tensile strength of the wood material, the tube will fracture, resulting in a tearing failure. If, instead, the compressive stress exceeds the compressive strength, the tube will collapse, resulting in a local compression failure. Wood is a natural orthotropic material with fibres aligned along the tree axis, and its compressive strength is about one-third of the tensile strength. Accordingly, local compression failure is expected to occur at a slightly lower bending moment than the tearing failure.

#### 2.1.2. Failure by Brazier buckling (*M*_B_)

**Figure 3.**
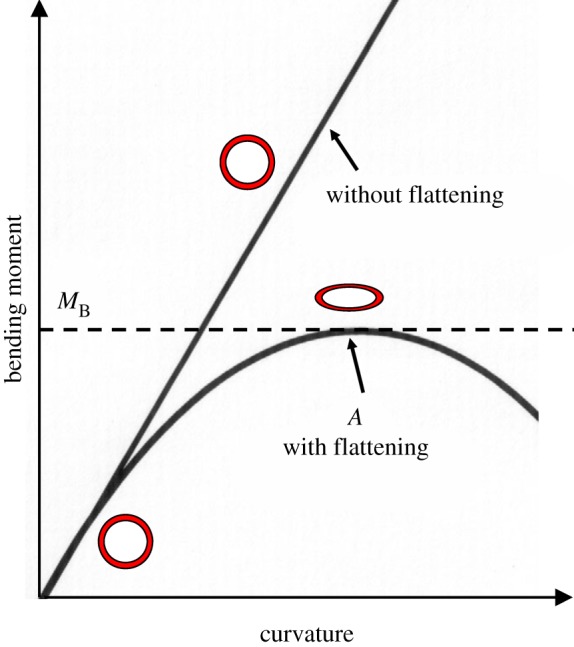
Relationship between the bending moment and curvature of thin hollow trunks with and without considering the cross-sectional flattening. The flattening occurs at *A* with a maximum bending moment *M*_B_, known as Brazier's critical buckling moment.

When a long, straight cylinder is bent, its curvature is proportional to the bending moment. Tensile stresses on the convex side and compressive stresses on the concave side both produce transverse compressive forces which increase with the curvature. The Brazier moment describes the effect of ovalization on the buckling of thin tubes. Brazier noted that buckling can occur in a thin-walled tube subjected to bending moment because the cross section ovalizes. An initially circular tube becomes oval, decreasing its second area moment and stiffness, and collapses as a result of structural instability. If the cross section is assumed unchanged during bending, the bending moment is proportional to the curvature as predicted by classic bending theory. In reality ovalization of the cross section may occur if the *t*/*R* is small, and the relationship between the bending moment and curvature becomes nonlinear (figure 3). When considering cross-sectional flattening, there exists a maximum bending moment *M*_B_ at which the tube becomes unstable and results in local kinking and collapse. This critical bending moment for isotropic materials is known as the Brazier buckling moment. Several authors [12,16–18] extend the Brazier moment for orthotropic materials as

2.2 $$M_{B}=\frac{2\sqrt{2}\pi R^{3}}{9}{\left(\frac{t}{R}\right)}^{2}\frac{\sqrt{E_{L}E_{T}}}{\sqrt{1-\nu_{\mathrm{LT}}\nu_{\mathrm{TL}}}}\approx \frac{2\sqrt{2}\pi R^{3}}{9}{\left(\frac{t}{R}\right)}^{2}\sqrt{E_{L}E_{T}},$$

where *E*_L_ and *E*_T_ are the longitudinal and tangential modulus of elasticity, respectively, *ν*_LT_ and *ν*_TL_ the Poisson's ratios. It is interesting to note that *M*_B_ is directly obtained by replacing the Young's modulus *E* and Poisson's ratio *ν* with the geometric average $\sqrt{E_{L}E_{T}}$ and $\sqrt{\nu_{\mathrm{LT}}\nu_{\mathrm{TL}}}$ in Brazier's formula [13].

#### 2.1.3. Failure by tangential cracking (*M*_c_)

**Figure 4.**
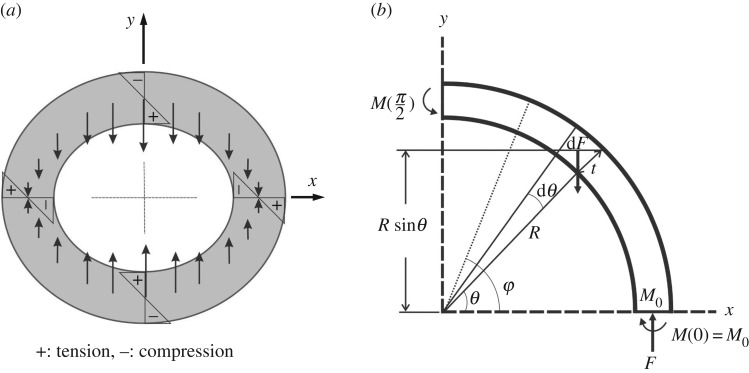
Analysis of cross-sectional flattening of a circular hollow trunk due to bending moment *M*_x_. (*a*) Inward force distribution in a transverse section. (*b*) Free-body diagram of one quarter of the transverse section for calculating the bending moment *M*(*φ*) exerted on axial sections with an angular position *φ*. *θ*, angular position of the force d*F*; *M*_0_, statically indeterminate bending moment at *φ* = 0. Note that the inward force d*F* is body force, not shear force, acting on the bulk of material.

The bending deformation of the tube induces longitudinal tensile strain/stress on the convex side and compressive strain/stress on the concave side of tube. The tensile stress and compressive stress both produce inward forces towards the *x*-axis (neutral axis), which tend to ovalize the cross section [2,19] (figure 4*a*). The inward force d*F* per unit axial length and a circumferential width of *R *d*θ* set up by the bending stress *σ*_L_ induced by a bent tube with a radius of curvature *ρ* is (figure 4*b*)

2.3 $$dF=\frac{1}{\rho }\sigma_{L}tR\;d\theta .$$

Note that d*F* is a body force, not shear force, acting on the bulk of material. Substituting for *ρ* and *σ*_L_ into equation (2.3), we obtain

2.4 $$dF=\frac{M_{x}}{E_{L}I}\;\frac{M_{x}}{I}R^{2}t\;\sin\theta \;d\theta ,$$

where *I* = *πR*^3^*t* is the moment of inertia of the circular cross section of a thin tube. Owing to symmetry only one quarter of the ring is considered. The total force per unit length *F* is expressed as

2.5 $$F=\int_{0}^{\pi /2}dF=\frac{M_{x}^{2}}{\pi^{2}E_{L}R^{4}t}.$$

Equation (2.4) is then rearranged as d*F* = *F* sin*θ *d*θ*.

The magnitude of the tangential bending moment *M*_0_ per unit length acting on the axial cross section at *φ* = 0 is statically indeterminate and can be calculated using Castigliano's theorem. By using *M*(*φ*) as the moment of unit length at an angle *φ* from the *x*-axis (figure 4*b*) and *I*_z_ as the moment of inertia of unit length of the cross section, the strain energy *U* per unit length for one quarter of the section is

2.6 $$U=\int_{0}^{\pi /2}\frac{M{\left(\varphi \right)}^{2}Rd\varphi }{2E_{T}I_{z}}$$

and the moment *M*(*φ*) is given by

2.7 $$\begin{array}{rl} M(\varphi ) & =M_{0}-\text{FR}(1-\cos\varphi )+\int_{0}^{\varphi }\text{FR}(\cos\theta -\cos\varphi )\sin\theta \;d\theta \\ & =M_{0}-\frac{1}{2}\text{FR}(1-\cos^{2}\varphi ). \end{array}$$

A positive *M*(*φ*) tends to increase the local curvature at *φ*. Owing to the condition of symmetry the cross section at *ϕ* = 0 does not rotate during bending, such that

2.8 $$\frac{dU}{dM_{0}}=0.$$

Because d*M*(*ϕ*)/d*M*_0_ = 1, we have

2.9 $$\frac{1}{EI_{z}}\int_{0}^{\pi /2}\left[M_{0}-\frac{1}{2}FR(1-\cos^{2}\varphi )\right]R\;d\varphi =0,$$

then

2.10 $$M_{0}=\frac{1}{4}FR.$$

Substituting for *M*_0_ from equation (2.10) into equation (2.7), we obtain

2.11 $$M(\varphi )=\frac{1}{2}FR\left(\cos^{2}\varphi -\frac{1}{2}\right)$$

and *M*(0) = *M*_0_ = *FR*/4; *M*(*π*/2) = −*FR*/4; *M*(*π*/4) = 0.

Using tangential bending strength *σ*_T_ the tangential bending moment at failure *M*_T_ can be expressed as

2.12 $$M_{T}=\frac{t^{2}\sigma_{T}}{6}.$$

Substituting for *M*(0) = *M*_T_ = *FR*/4 and *F* from equation (2.5) into equation (2.12), we obtain longitudinal bending moment *M*_c_ at which the tangential cracking is initiated at *φ* = 0

2.13 $$M_{c}=\pi R^{3}\sqrt{\frac{2E_{L}\sigma_{T}}{3}}{\left(\frac{t}{R}\right)}^{3/2}.$$

Note that equation (2.13) is the most important result of this study; it is derived for the first time and has not been reported in the literature. The distribution of normal stresses in the axial section at *φ* = 0 can be obtained by superposing the stress (*σ*_1_) due to the normal force *F* and the stress (*σ*_2_) due to the bending moment *M*_0_ = *FR*/4. The ratio of these two components is $\sigma_{2}/\sigma_{1}=(6M_{0}/t^{2})/(F/t)=3R/2t\gg 1$ , indicating that *σ*_1_ is negligible when R≫t.

Note that the maximum absolute value of *M*(*φ*) occurs at *φ* = 0 and *π*/2, and *M*(0) = −*M*(*π*/2) = *FR*/4. Hence when the applied bending moment *M*_x_ exceeds *M*_c_, tangential cracking, followed by longitudinal splitting, may occur simultaneously at the four vertices of the oval ring, and the cracking occurs on the inner surfaces at *φ* = *π*/2 and 3*π*/2, and outer surfaces at *φ* = 0 and *π*.

#### 2.1.4. Failure by longitudinal shear stress (*M*_s_)

An alternative explanation for longitudinal cracking was suggested by Mattheck *et al*. [7], who emphasized that splitting can result from shear stresses in the axial direction. Consider a hollow cylindrical trunk as a cantilever loaded by a wind load *P* at its crown. The corresponding internal bending moment at a distance *h* from the centroid is *M*_x_ = *Ph* (figure 2), and the maximum shear stress *τ*_max_ at the neutral axis is [19]

2.14 $$\tau_{\max}=\frac{4P[1+(1-t/R)+{\left(1-t/R\right)}^{2}]}{3\pi R^{2}[1-{\left(1-t/R\right)}^{4}]}.$$

Substituting shear strength *τ*_s_ and *M*_s_/*h* for *τ*_max_ and *P* in equation (2.14), we obtain the breaking moment for shear

2.15 $$M_{s}=\frac{3\pi hR^{2}\tau_{s}[1-{\left(1-t/R\right)}^{4}]}{4[1+(1-t/R)+{\left(1-t/R\right)}^{2}]}.$$

#### 2.1.5. Critical *t***/***R* ratios

Critical ratios (*t*/*R*)_cB_, (*t*/*R*)_Bb_ and (*t*/*R*)_cb_ that determine the failure mode at a given *t*/*R* ratio are calculated by setting *M*_c_ = *M*_B_, *M*_B_ = *M*_b_ and *M*_c_ = *M*_b_, respectively, using $M_{b}=\pi R^{3}(t/R)\sigma_{b}$, equations (2.2) and (2.13) as follows:

2.16 $$\begin{array}{rl} {\left(\frac{t}{R}\right)}_{\mathrm{cB}} & =\frac{27\sigma_{T}(1-\nu_{\mathrm{LT}}\nu_{\mathrm{TL}})}{4E_{T}}\approx \frac{27\sigma_{T}}{4E_{T}}, \end{array}$$

2.17 $$\begin{array}{rl} {\left(\frac{t}{R}\right)}_{\mathrm{Bb}} & =\frac{9\sigma_{b}\sqrt{1-\nu_{\mathrm{LT}}\nu_{\mathrm{TL}}}}{2\sqrt{2}\sqrt{E_{L}E_{T}}}\approx \frac{9\sigma_{b}}{2\sqrt{2}\sqrt{E_{L}E_{T}}}, \end{array}$$

2.18 $$\begin{array}{rl} {\left(\frac{t}{R}\right)}_{\mathrm{cb}} & =\frac{3\sigma_{b}^{2}}{2E_{L}\sigma_{T}}. \end{array}$$

The equations derived in this section are applicable to analyse straight hollow trunks of tree species and bamboo stems, but are not applicable to those with side openings or those with only heart decay. Trunks with heart decay are more resistant to flattening failure; the theoretical analysis of hollow trunks with side openings is more complicated and is beyond the scope of this paper.

### 2.2. Experimental demonstration of tangential cracking

**Figure 5.**
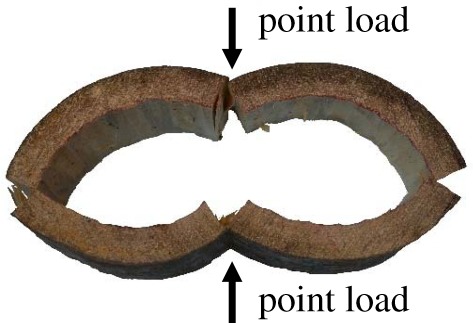
Experimental result of longitudinal splitting of a hollow trunk of softwood (*Araucaria cunninghamia*) subjected to two opposite compressive point loads, which may be used to mimic the failure mode of tangential cracking due to cross-sectional flattening during bending. However, it is important to note that the loading here is not a realistic representation of the internal force generated by flattening during bending, and hence its bending moment at cracking given in [12] is expected to deviate from equation (2.13) derived using more realistic loading condition in this study.

To experimentally illustrate the failure appearance of tangential cracking, we conducted computer-controlled compression tests (HT-9102, Hung Ta Instrument Co. Ltd.) on a hollow trunk of softwood (*Araucaria cunninghamia*). The hollow section, artificially created from a solid one with *t* = 30 mm and *R* = 125 mm (*t*/*R* = 0.24), was subjected to vertical point loads, and failed due to cross-sectional flattening (figure 5). We repeated the same experiment twice on *A. cunninghamia* and bamboo stems (*B. oldhamii*), and observed the same failure mode—cracking, followed by splitting---began on the outer surfaces of the left and right ends, and on the inner surfaces of the top and bottom positions, where the maximum tensile stresses are located. This failure appearance well corresponds to the tangential cracking predicted by our analysis in §2.1.3, and is consistent with earlier work by Mattheck [20] and Spatz & Niklas [2]. However, the loading here is external and is different from that which a tree really experiences during bending despite their similar failure appearance. The formula, based on external point loading [12], is expected to introduce some deviation from the actual case. On the other hand, equation (2.18) does not assume external point loading and is more accurate in predicting the critical *t*/*R* ratio.

## 3. Results and discussion

**Figure 6.**
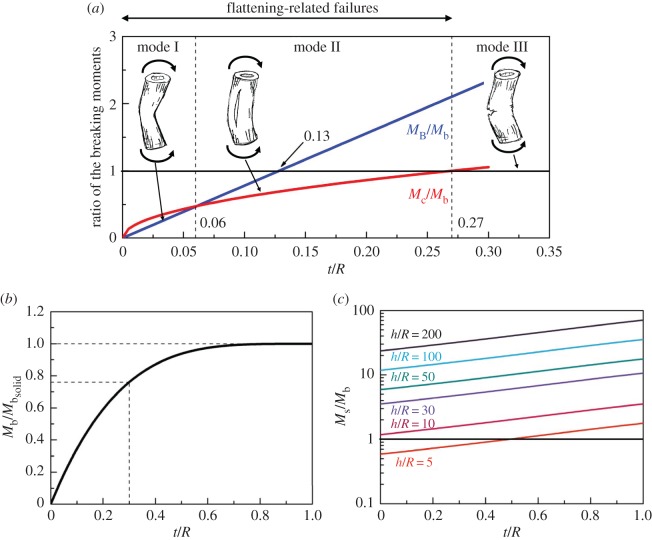
Ratios of the bending moment of different failure modes as a function of *t*/*R*. (*a*) The dominant failure modes are Brazier buckling (Mode I), tangential cracking/longitudinal splitting (Mode II) and conventional bending failure, i.e. compression failure on the compression side and tearing on the tension side (Mode III) for 0 < *t*/*R* < 0.06, 0.06 < *t*/*R* < 0.27 and 0.27 < *t*/*R* < 1, respectively. (*b*) Effect of hollowness on the conventional bending failure. A hollow trunk with *t*/*R* = 0.3 can resist 76% of a solid trunk's failure bending moment. (*c*) Comparison of shear failure (*M*_s_) and bending failure (*M*_b_) versus *t*/*R* and slenderness *h*/*R*. Conventional bending failure is more dominant for trees with *h*/*R* > 10, whereas shear failure may only occur for extremely small *h*/*R* ratio and thin-walled trunk. (Trunk sketches by Da-Chang Yang).

In this section, we describe our case studies of four tree species using the equations presented in §2. Their mechanical properties and critical *t*/*R* ratios are summarized in table 1. Figure 6*a* shows the failure moment ratios as a function of *t*/*R* for Taiwan red cypress (*Chamaecyparis formosensis*). Three dominant failure modes and their corresponding *t*/*R* ranges are determined as Brazier buckling (Mode I, 0 < *t*/*R* < 0.06), tangential cracking/longitudinal splitting (Mode II, 0.06 < *t*/*R* < 0.27) and conventional bending failure, i.e. compression failure on the compression side and tearing on the tension side (Mode III, 0.27 < *t*/*R* < 1). The critical ratios (*t*/*R*)_cB_, (*t*/*R*)_Bb_ and (*t*/*R*)_cb_, calculated from equations (2.16–2.18), are 0.06, 0.13, and 0.27, respectively. Among them, Mode I and Mode II involve cross-sectional flattening and Mode III does not. Although a critical ratio of *t*/*R* = 0.13 exists for Brazier buckling, it is unlikely to occur since the breaking moment for tangential cracking *M*_c_ is much smaller.

**Table 1.** Mechanical properties and critical *t*/*R* ratios of four green wood species. The mechanical properties of *Chamaecyparis formosensis* are from [21]; the *E*_L_ and *σ*_b_ values for *Araucaria cunninghamia*, *Terminalia boivinii* and *Lithocarpus castanopsisifolius* were measured by static bending test in our laboratory; *σ*_T_ = *σ_b_*/20 for softwood and *σ*_T_ = *σ*_b_/15 for hardwood [22]; *E*_T_ = *E*_L_/20 [23].

	Young's modulus (GPa)		bending strength (MPa)				
species	*E*_L_	*E*_T_	*σ*_b_	*σ*_T_	(*t*/*R*)_cB_	(*t*/*R*)_Bb_	(*t*/*R*)_cb_
*C. formosensis*^a^	4.9	0.25	45	2.2	0.06	0.13	0.27
*A. cunninghamia*^a^	9.9	0.50	71	3.5	0.05	0.10	0.21
*T. boivinii*^b^	7.1	0.36	77	5.1	0.10	0.15	0.24
*L. castanopsisifolius*^b^	8.8	0.44	82	5.5	0.08	0.13	0.21

^a^Softwood.

^b^Hardwood.

The failure mode of the other three species, as a function of *t*/*R* ratio, is similar to that of Taiwan red cypress (table 1). Their (*t*/*R*)_cb_, the most important results of this work, range from 0.21 to 0.27. Our prediction of a tangential cracking at *t*/*R* < 0.27 is in remarkable agreement with the well-appreciated field observations conducted by Mattheck & Breloer [20]. They examined over 1000 broken and standing hardwood and softwood trees in parks and along roadsides, and found that most of the broken trees had *t*/*R* values < 0.3. Therefore, the ratio *t*/*R* = 0.3 has been used worldwide for VTAs as an approximate criterion for the upper critical limit in risk evaluation of decayed trunks. This excellent agreement indicates that our analysis captures the physics of the failure mechanism of trees.

Hollow tubes have the advantages of resisting bending and torsional moments with a relatively lower weight per unit length than solid cylinders of the same weight. A hollow trunk of *t*/*R* = 0.3 still contains a residual bending strength (*M*_b_) of 76% of a solid one $\left(M_{b_{\mathrm{solid}}}\right)$ with the same outer radius (figure 6*b*). If we assume that a sound trunk has a safety factor of four [7], the safety factor of a hollow trunk with a *t*/*R* of 0.3 is three. However, the ratio $M_{b}/(M_{b_{\mathrm{solid}}})$ drops significantly when *t*/*R* < 0.3, which is compounded by the cross-sectional flattening and makes those trees particularly vulnerable to bending.

Broken trees of *t*/*R* ratio below 0.05 are almost non-existent, suggesting that failure by Brazier buckling is indeed very rare in the field. Accordingly, tangential cracking followed by longitudinal splitting becomes the dominant failure mode of hollow trunks. Wegst & Ashby [12] also derived equations to calculate the bending moment for longitudinal splitting. They assumed external point loads similar to our experimental condition (figure 5). Those equations, although originally derived for plants with hollow stems, have been adopted to analyse mechanical failure in animal exoskeletons and endoskeletons [3]. We find that their formula for *M*_c_ at *φ* = 0 gives very similar results to equation (2.13) (less than 10% deviation for 0.1 < *t*/*R* < 1). However, due to the assumed external point loads, their formula predicts a much smaller *M*_c_ at *φ* = *π*/2 (approx. 73% of the *M*_c_ at *φ* = 0), meaning that the tangential cracking will always initiate at the load application points, i.e. *φ* = *π*/2 and 3*π*/2. By contrast, our analysis does not assume external point loads and predict that the tangential cracking initiates simultaneously at the four vertices of the oval ring. On the other hand, Spatz & Niklas [2] conducted comprehensive computer simulations to estimate the breaking moments for different failure modes. They assume that 10% ovalization leads to Brazier buckling, and critical failure strains are 0.3 and 2% in the axial and tangential directions, respectively. Their simulation results of (*t*/*R*)_cB_ = 0.11 and (*t*/*R*)_cb_ = 0.23 are remarkably close to those obtained by our formula, although different material properties are used (figure 6*a*). Besides, their simulation showed that cross-sectional flattening only plays a role for *t*/*R* < (*t*/*R*)_cb_, which is also consistent to our analysis.

An alternative explanation of longitudinal splitting is due to longitudinal shear stress, proposed by Mattheck *et al*. [7]. They reported that trunk failure begins with a shear crack combined with flattening of the cross section, followed by bending failure, and suggested that complex effects of shear, bending stress, longitudinal splitting and the Brazier moment should be considered in determining the mechanism of trunk breakage. Here we assume *σ*_b_ = 8.5 *τ*_s_ [22] and compare the magnitudes of bending moments, required to initiate shear failure and bending failure, by calculating *M*_s_/*M*_b_ versus *t*/*R* and slenderness ratio *h*/*R*. *M*_s_/*M*_b_ ratios increase with both *t*/*R* and *h*/*R.* Conventional bending failure is more dominant for trees with *h*/*R* > 10, whereas shear failure is only possible for extremely small *h*/*R* and thin-walled trunk (figure 6*c*). Spatz & Niklas [2] simulated the ratio of compressive stress to shear stress versus *t*/*R* and *h*/*R*, and also concluded that shear failure is unlikely to occur except for very short and thin-walled trunks. Nevertheless, by using finite-element method, Mattheck *et al*. [7] found that the ‘crossing’ of tensile and compressive stresses at the tree butt may significantly amplify the shear stress and initiate axial shear cracks even in solid trunks. This is an interesting viewpoint worth further research.

Growth stresses are ubiquitous in trees and may affect the failure mechanism. Longitudinal growth stress of normal wood is tensile on the surface of the trunk and is compressive inside [21,24,25], whereas tangential growth stresses are always compressive on the surface but tensile inside. Because the longitudinal compression strength of wood is only about one-third of its tensile strength, longitudinal tensile growth stresses can partially compensate for the compressive stresses on the leeward side of a trunk induced by the bending moment of wind loads acting on its crown. The compressive surface growth stress in the tangential direction can partially resist longitudinal splitting. These features may be recognized as the self-optimized design of trees as a result of evolution.

Hazard assessments of hollow trunks with respect to flattening failure can be achieved by comparing the Brazier moment, tangential splitting moment and bending failure moment with the wind load moment. To calculate the wind load moment, the wind speed of a hurricane (33 m s^−1^, Beaufort scale 12) is usually adopted as a standard. The crown area and centroid height can be estimated by image analysis. If the wind load moment is larger than these moments, it is possible that a hollow tree may fail.

## 4. Conclusion

In this paper, we investigate the failure mechanism of hollow trunks due to bending by comparing various failure modes, including Brazier buckling, tangential cracking/longitudinal splitting, conventional bending failure and shear failure. We derive a new analytical expression for predicting the bending moment at which tangential cracking occurs, and show that the cracking, followed by longitudinal splitting, is initiated simultaneously at the four vertices of the ovalized section. We use Taiwan red cypress as an example, and find that its dominant failure modes are Brazier buckling (Mode I), tangential cracking/longitudinal splitting (Mode II) and conventional bending failure (Mode III) for 0 < *t*/*R* < 0.06, 0.06 < *t*/*R* < 0.27 and 0.27 < *t*/*R* < 1, respectively. Among those, Mode I and Mode II are flattening-related failures. The critical value of (*t*/*R*)_cb_ = 0.27 indicates that trees with a *t*/*R* < 0.27 tend to fail with longitudinal splitting, which is in remarkable agreement with the well-appreciated criterion of VTAs. Our analysis provides new insights into the understanding of hollow tree failures, in particular the origin of longitudinal splitting. Our findings may be readily applied to assess the failure potential of other tree species with different material properties. Our theoretical framework is applicable to analyse straight hollow tree trunks and plant stems, but is not applicable to those with side openings or those with only heart decay.

## Supplementary Material

Source data

## Data accessibility

The datasets for generating figure 6 have been uploaded as the electronic supplementary material alongside this article.
